# Automatic Calibration and Update of a Digital Twin for Plug & Produce

**DOI:** 10.3390/s25226885

**Published:** 2025-11-11

**Authors:** Mattias Bennulf, Sudha Ramasamy, Xiaoxiao Zhang, Fredrik Danielsson, Janardhanan Swathanandan

**Affiliations:** 1 Department of Engineering Science, University West, 461 86 Trollhättan, Swedenxiaoxiao.zhang@hv.se (X.Z.); fredrik.danielsson@hv.se (F.D.); 2 GKN Aerospace Engine Systems, 461 81 Trollhättan, Sweden; swathanandan.janardhanan2@gknaerospace.com

**Keywords:** Industry 4.0, digital twin, calibration, manufacturing, industrial robots, Plug & Produce

## Abstract

**Highlights:**

**What are the main findings?**

- A digital twin, consisting of an automated path planner and a simulation model, is proposed for an industrial robot to automatically adapt to new locations of resources that have been moved.
- The system should update the digital twin with calibrated values of the resource locations due to the tight tolerances in the processes and physical limitations of placing resources out precisely.

**What are the implications of the main findings?**

- Using the proposed system, the manufacturing can still function if resources are moved around frequently.
- The camera-based solution for automating the measurement of resource locations was implemented in a physical demonstrator.

**Abstract:**

This article presents a system for automatically updating a digital twin model, used for automated path planning of an industrial robot. The digital twin needs to be accurately calibrated in relation to the resource locations due to the physical limitations of placing resources out precisely. The process considered is a surface roughness measurement of aerospace metal parts that requires high positional accuracy. The scenario takes place in a robot cell that is a Plug & Produce system, where resources can be added and removed in minutes, allowing fast reconfiguration of the production resources. This means that an automated path planner is required for the robot to adapt to new locations of these resources automatically. A digital twin is proposed, consisting of a robot path planner and a simulation model that is updated when resources are added to the system. The resources should automatically appear in the simulation and be placed at an accurate location. The purpose of automating these steps is to make the update of the digital twin faster during production and remove the requirement for expert knowledge.

## 1. Introduction

A shift from mass production to mass customisation is a necessary step for Industry 4.0 to keep up with customer demands for customised or low-volume manufacturing [1,2]. Rebuilding manufacturing systems typically takes several months to complete and is related to high costs. Flexible approaches, such as Plug & Produce (P&P) systems that can quickly be repurposed rather than replaced or rebuilt, are a necessary step for reaching faster adaptation times. This concept was first introduced by Arai et al. [3] This article focuses on a P&P system where resources can be reused for different manufacturing scenarios using standardised interfaces for connecting them at different locations.

**Figure 1:**
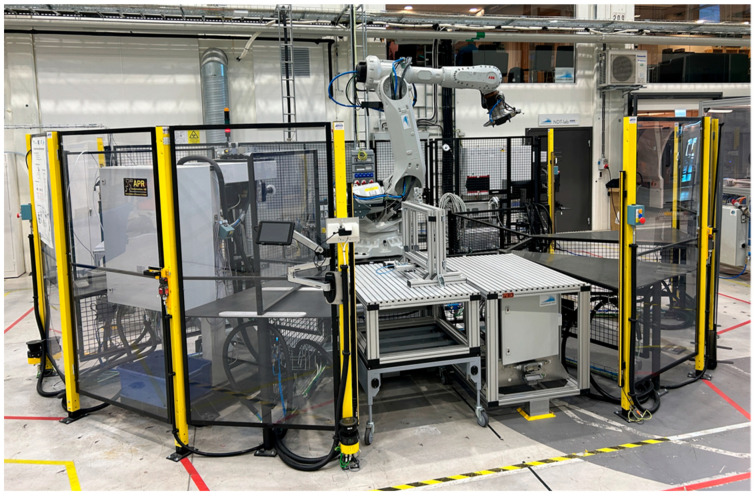
The “P&P test bed”, located in Trollhättan Sweden, that was used for the experiment and for the manufacturing scenario considered.

A P&P-based robot cell located at the University West robotic lab in Trollhättan Sweden, was used for the experiments in this article. It will be referred to as the P&P test bed in this article. This cell was presented in detail in [4,5]. A system that can be adapted fast to changes needs to consider the time to change hardware and the time to change the software. The hardware time can be improved by implementing modules [6,7]. The software time requires reprogramming or reconfiguration of the logical system to adjust for added resources [8]. Another concept is Reconfigurable Manufacturing Systems (RMS), and there exist many examples in the literature, such as [9]. However, existing systems still have too-high installation costs for use with low-volume production and mass customisation [10,11,12]. Thus, the P&P test bed consists of multiple process modules that are 800 times 1200 millimetres (the size of a euro pallet), see Figure 1. Each process module contains some type of process that can be performed in the robot cell. An industrial robot is placed in the centre of these modules so that it can reach all of them. Modules can be used for many things, such as loading and unloading parts from the cell, storing parts, and processes such as drilling, measuring, and grinding. The process modules can be moved around to any slot in the robot cell since they all have standardised physical and logical interfaces. This also makes it possible for them to be placed in storage temporarily, when not needed or moved to other robot cells. The process modules are made of aluminium profiles and are attached to the cell by metal aligners that guide the module to be placed in the same location each time. This means that a coordinate system can be measured for each location of the process modules and be reused for any process module that is placed in that location. In some specific scenarios, the accuracy of the 3-2-1 position alignment is not enough to meet the process needs. When looking at the combined error in all three axes (x, y, z), an offset of more than 1000 microns can be observed even when a module is plugged into the same slot, which leads to low precision. Thus, a Work-object ($W_{obj})$ calibration is needed to minimise the positional errors in the translation and rotation axes.

Due to the flexible nature of the system described, robot paths cannot be static for all scenarios. Each process module can have many target locations that a robot should move between. The high number of possible combinations of moving between all locations in the P&P test bed requires the paths to be automatically generated. Manually creating all combinations would be far too time-consuming. Recent reviews of different path planning algorithms highlight that classical algorithms like A* and Dijkstra remain foundational due to their reliability and simplicity, especially in static environments [13], and modern approaches such as genetic algorithms, swarm intelligence, and machine learning-based methods are gaining traction for dynamic and complex scenarios [14]. The automated path planning systems described in [15,16], both using RobotStudio, are considered for the dynamic environment in the presented scenario. These advanced techniques offer adaptability and optimisation but often require higher computational resources. The choice of algorithm depends on factors like robot geometry, environment type, and real-time constraints. To make such a path planner completely automated, the calibration values for the process module locations need to be updated automatically when the system is running. Thus, this article focuses on the automated update of the simulation used for the path planner. This enables real-time adjustments, maximises productivity, minimises downtime, and reduces the need for manual interventions. This results in saving time as well as labour costs and enhances overall performance in manufacturing operations. In Figure 1, the P&P test bed is shown, which was used for the experiment and as a scenario for developing the proposed system.

This article explains a system that uses automatic measurements of each process module location in the P&P test bed. The accuracy needs to meet the requirements of a scenario where the surface roughness is measured on metal parts, with a 300 microns accuracy. Measuring surface roughness is conducted with a wireless profilometer mounted on an industrial robot. The calibration of the process module is implemented and tested using a camera and an asymmetric circle grid. Further, the article presents a complete system for updating a digital twin. This includes placing the computer-aided design (CAD) models of each process module in a digital twin based on the calibrated location values. This makes it possible for a path planner to automatically find a collision-free optimal path even when the P&P test bed has changed its set of process modules or moved them around.

## 2. Related Work

This chapter explains the related work on path planning, digital twins, and calibration approaches. Path planning can be conducted online or offline, where the online algorithms do not know the environment before running [17,18]. Automated path planning offline can help reduce labour costs and speed up the time to adapt manufacturing to new tasks. However, when products are manufactured in low volume or the product design is completely customised, human workers are usually introduced. This is because they are faster to adapt to new specifications. Using an online path planner, robots become more useful for such flexible industrial environments. To make this work, an updated virtual environment based on real-time sensor data is required [19]. The virtual environment is used to perform collision detection and to test the robot’s reachability. Another approach, explained by Arents et al. [20], is using input from a vision system. This allows locations of objects to be found and paths to be generated. Dzedzickis et al. [21] explained that path planning can be divided into global path planning, which means the system has in-depth knowledge about the navigation environment and local path planning, where limited knowledge is given about the environment.

A digital twin is important for online path planning since it provides an updated virtual environment for planning the paths. Zhang et al. [22] described a digital twin for an industrial robot that adapts to the environment automatically. Specifically, human–robot collaboration was investigated. It used the path planning software MoveIT to generate paths. Zhu et al. [23] described a digital twin that has access to physical geometric data, physical perception, process state, and simulation verification. This is conducted by connecting a virtual and physical robot together. Duan et al. [24] presented a system that also uses MoveIT. The system has a digital twin that has a physical and virtual space and a model fusion. The physical space contains equipment measurements, pose calibration, and space observations. The virtual space is used for visual presentation and virtual manipulation. The model fusion is used to connect the physical and virtual spaces.

The sensor-based approach achieves accurate motion control by providing real-time feedback based on actual measurements [25]. However, the limitations are that this method affects the compensation process, which is generally localised around the point of measurement. This means that when a robot moves away from this specific pose, the effectiveness of the compensation decreases due to discrepancies between the nominal kinematic model and the actual behaviour of the robot. Due to the robustness of this method and low investment cost, proximity sensor-based calibration is employed. The proximity sensor provides the positional calibration, and it can include compensation for the robot-specific absolute positioning error against the $W_{obj}$. This enhances the accuracy, and the calibration procedure can be directly integrated into the robot programming. It has the disadvantage of a longer cycle time, it can be identified only for a few objects, it requires a larger work area, and it consumes a lot of time for complicated geometries [26,27].

Different alternative approaches are incorporated in calibrating the $W_{obj}$ automatically. The first one is a fixed camera mounter around the robot’s workspace, and the second one is a local positioning module based on the camera mounted on the robot arm. This method can be applied only where a 1 mm tolerance is accepted [28]. Another low-cost and easily accessible $W_{obj}$ calibration method is proposed [29] with a touch panel. Here, the robot can make the cell calibrations and calculations by monitoring the touching status, but there is an issue with communication delay, and the resolution of the touch panel also affects the efficiency of measuring accuracy.

Huang et al. proposed a method using visual servo control that provides better accuracy, enables quick and efficient calibration of the $W_{obj}$ frame, potentially completing the process in just one minute, and can adapt to different $W_{obj}$ configurations and variations, providing flexibility in calibration processes, but fine-tuning the visual servo control system and the integration with image sensors makes it very complex [30]. Another method with two laser sensors is used, i.e., one in the X-axis direction and one in the Y-axis direction. Using these two sensors, the precise position of the Tool Center Point (TCP) is determined by finding the end of the tool along the axial direction and the homogeneous transformation matrix is calculated based on the sensor information. The main drawbacks are that traditional calibration methods suffer from poor accuracy and stability and strong operational dependence, which leads to limited practical applicability in industrial settings [31]. In another approach, two pairs of laser beam sensors are vertically mounted, and the robot moves the tool in a uniform and circular motion at various heights. Each time the tool passes the laser sensor, the position of the robot flange is recorded, and the position of the tool frame is identified [32]. The main drawback of using two laser sensors in that the tool frame involves huge investment.

Camera-based $W_{obj}$ calibration ensures the precise positioning and alignment of objects, thereby enhancing manufacturing efficiency and product quality. Moreover, it offers flexibility in adapting to different environments and setups and is cost-effective in the long term due to reduced downtime and manual intervention.

## 3. Proposed System

Even though it works well for many scenarios, the P&P test bed has some accuracy problems noted when modules have been detached and attached again, even to the same location, due to the tolerances in the physical attaching mechanism. Improvements could be considered for the physical system, but instead, this article proposes a system that uses a camera to accurately measure the process module location after being attached to the P&P test bed.

Each process module is considered a local controller that we regard as an agent in the system. An agent is a software that can be instructed with high-level goals that are reached by communicating with other agents [33]. This means that all knowledge about each process on the modules is stored in the modules and not in the industrial robot or other central controllers, as in traditional systems. The robot instead has a local behaviour only to take locations and move to them, attach and detach tools, and trigger tool functions, such as open and close the vacuum. Robot tools have local behaviours as well, knowing what the tool can perform. Using this type of distributed approach is called a multi-agent system. It means that resources only have knowledge that is needed locally and can easily be moved to other systems without any reprogramming. The local controller can also be placed in a cloud service if a local computer is not available. Each part to be processed in the system has goals it wants to achieve and uses the resources to reach those goals. One example of such a type of multi-agent system is the Configurable Multi-Agent System (CMAS) described in [5].

**Figure 2:**
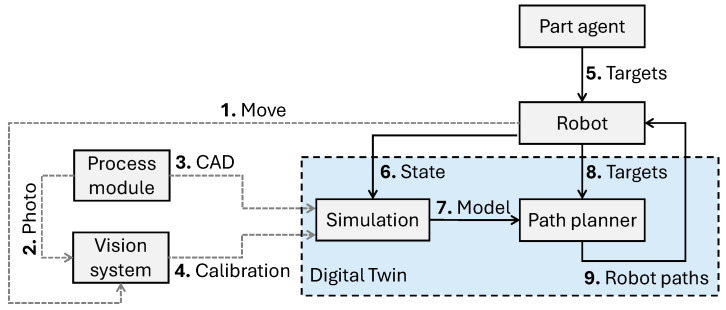
The proposed system for automatic update of the digital twin model.

The agents needed for the scenario are a part agent, a robot agent, a process module agent, a digital twin agent, and a vision system agent. These agents represent the actual hardware and software shown in Figure 2. In CMAS, all agents run on one computer and communicate with each other in that environment to negotiate and share data. There are several standard communication protocols available in CMAS for the connection between agents and the object they represent, except for the part agent that has no physical computer on it.

The rest of this chapter describes the procedure of installing a process module in the robot cell, how to calibrate it, update the digital twin, and generate a robot path to run. First, the process module is installed physically and connected to the network and power. This triggers a calibration sequence, shown as grey dashed lines in Figure 2. When the system is calibrated, it runs as described by the black solid lines in Figure 2.

The calibration value is the deviation from the values of an initial coordinate system of the process module, which is measured using a vision system when the system is considered calibrated. For example, when target locations were defined on the process module using the robot.

**Figure 3:**
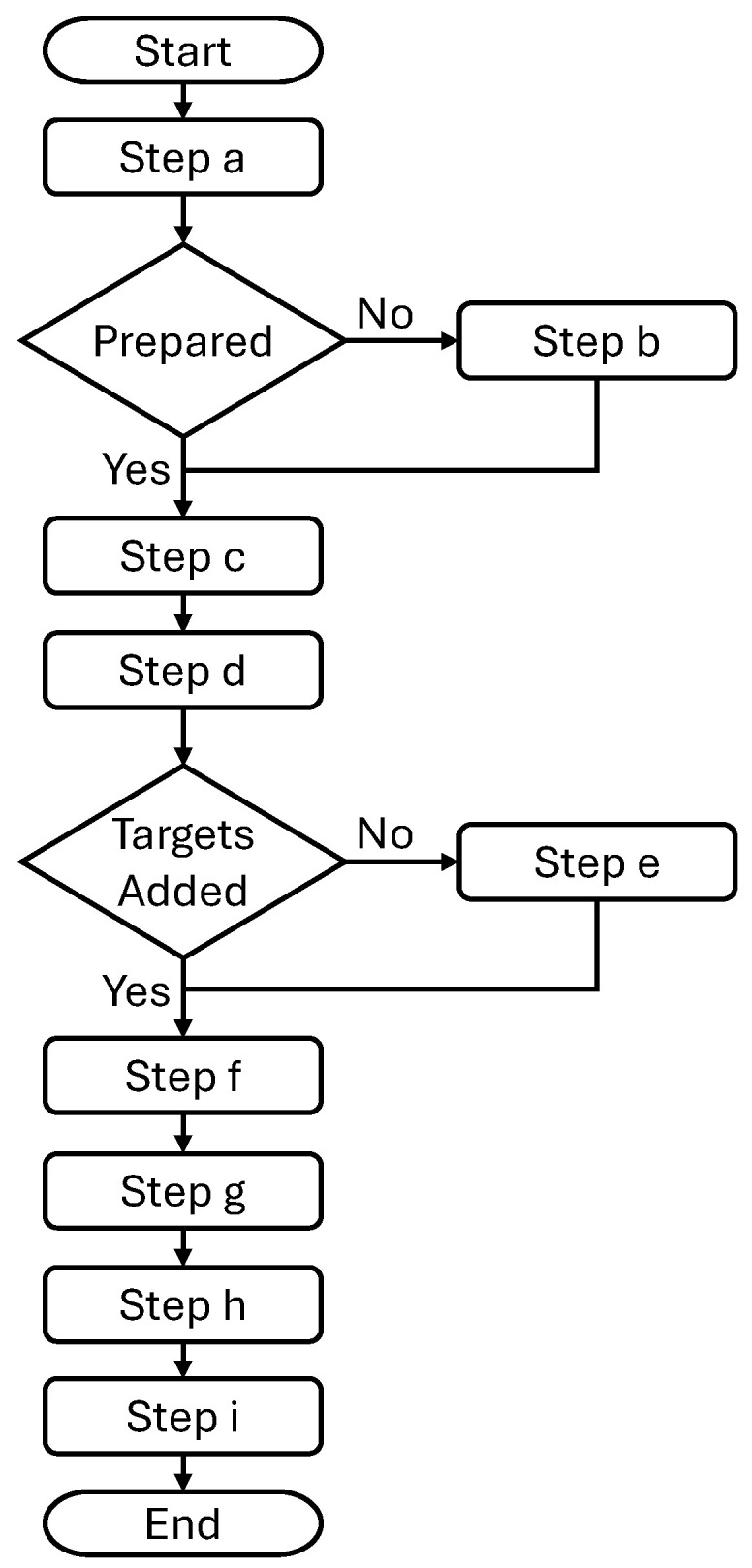
The flow of running the steps of the manufacturing scenario.

The following list explains the scenario steps a, b, …,i of the manufacturing scenario that is considered for the proposed system for automatic update of the digital twin model. This is also illustrated as a flowchart in Figure 3. The list also includes descriptions of the actions 1, 2, …, 9 shown in Figure 2.

Step (a) The process module is physically installed in the system, taking a few minutes using standardised connectors.
Step (b) If not already conducted, the process module is prepared manually with a CAD model and an agent to represent the module. All data is stored in the physical process module.
Step (c) The robot is requested to start a sequence to move (1) the vision system (camera) to an asymmetric circle grid that is mounted on the process module.
Step (d) A photo (2) is taken of the circle grid, and the vision system uses its computer to calculate the location of the process module.
Step (e) If not already conducted for this process module, target locations for the robot to move to are defined and stored in the part agent. For example, locations for scanning a metal part or pick and place locations.
Step (f) When a part agent in the system requests the robot to move, the CAD models (3) are automatically copied to the simulation in the digital twin at the locations that are adjusted with the calibration values (4).
Step (g) The targets (5) are sent to the robot, and the robot sends its state (6) to the simulation.
Step (h) The path planner generates a collision-free path for the robot based on the simulation model (7) and the target locations (8) that the robot should move to.
Step (i) The robot receives the robot paths (9) and runs them, completing the defined process.

### Updating Digital Twin

**Figure 4:**
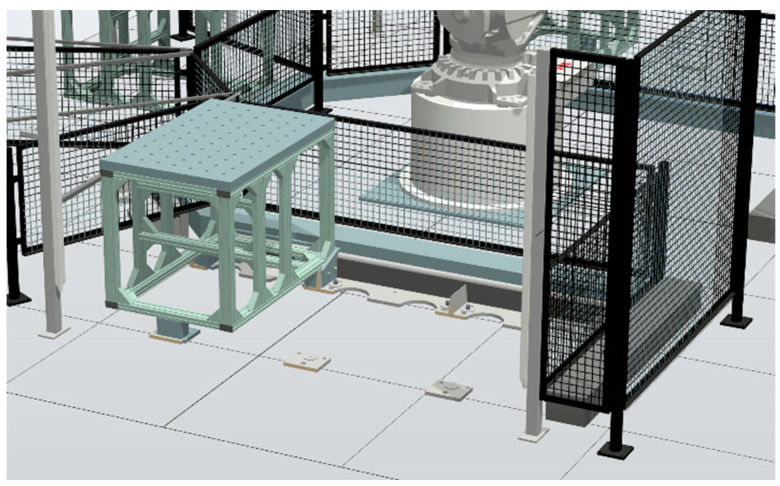
Simulation model for the digital twin with three slots shown for adding process modules.

To update the digital twin, the simulation model running in RobotStudio, shown in Figure 4, is considered. This shows an example of placing a process module in the simulated P&P system. It has previously been tested in [4] to detect process modules and connect them to the P&P demonstrator considered. Using the SDK for RobotStudio, add-in software can be developed in the C# programming language. This can be used to build an add-in that automatically reads CAD model data and a JSON file including the file name of the CAD model, path to the directory, position x, y, z, and orientation rx, ry, rz. The vision system is considered to be an agent that communicates the calibration values formatted as JSON to the digital twin agent. The SDK has functions that can be used to load the models to the correct location based on that data. The add-in can also be designed to connect to a multi-agent system of the type CMAS, presented earlier in this chapter. This is possible since CMAS has several standard communication protocols that can be connected to from the add-in, such as Modbus, Open Platform Communications Unified Architecture (OPC UA), and an Application Programming Interface (API) for Representational State Transfer (REST), commonly termed RESTful API. This will enable the add-in to read the robot’s state and update the robot in the simulation.

## 4. Implementation and Results

This chapter explains the implementation and results. The focus of this article is the *proposed system* that connects several systems together. Some of these systems have already been tested individually in previous work. The agent system CMAS [5] and the process module identification system in [4] have both been tested previously with the P&P demonstrator considered. The automated path planning system considered has been tested in [15,16], which uses RobotStudio for collision detection of the robot. Thus, the experiment was focused on automating the camera-based vision system and setting it up so that it can easily be connected with the digital twin.

### 4.1. Vision System Implementation

This section explains the details of how the vision system was implemented and connected to the P&P test bed. A vision-based tool has been developed to detect the displacement of process modules.

There were seven requirements for the setup of the vision system. The camera system (1) consists of an industrial camera from Allied Vision Technologies (Alvium 1800) and a lens that can adjust the aperture and focus. The image acquisition (2) was conducted using the Vimba API to connect to the camera. We turned off the auto exposure and auto gain and set the exposure and gain manually according to the illumination in the physical setup. The robot system (3) consisted of an industrial robot ABB IRB6700, installed with a safety system next to the process modules. For the calibration pattern (4), a professionally printed circle grid, printed on a hard surface to avoid warping, was used. To be used as a camera computer (5), an Intel Joule 570x running Linux OS was selected. It was mounted on the robot and connected to the camera. The vision library (6) selected was the Open-Source Computer Vision Library (OpenCV), mainly using the function “solvePnPRansac”, running on the Intel Joule computer to detect the location of the circle grid. The network connection (7) was implemented using a RESTful API on the Intel Joule computer.

The vision-based tool assumes that the transformation between the asymmetric circle grid and the robot target locations is static since the modules are rigid bodies. Thus, the deformation of the module material is not considered while calibrating the position.

Modelling the transformation in $SE\left(3\right)$ and assuming the 3D homogeneous transformation technique of $SE\left(3\right)$:

(1) $$SE\left(3\right)=\left\{T\;\vdots \;T=\left[\begin{array}{cc} R & r\\ 0_{1\times 3} & 1 \end{array}\right],\;R\in R^{3\times 3},\;r\in R^{3},\;R^{-1}R=RR^{-1}=Ι,\;\left|R\right|=1\right\}$$

**Figure 5:**
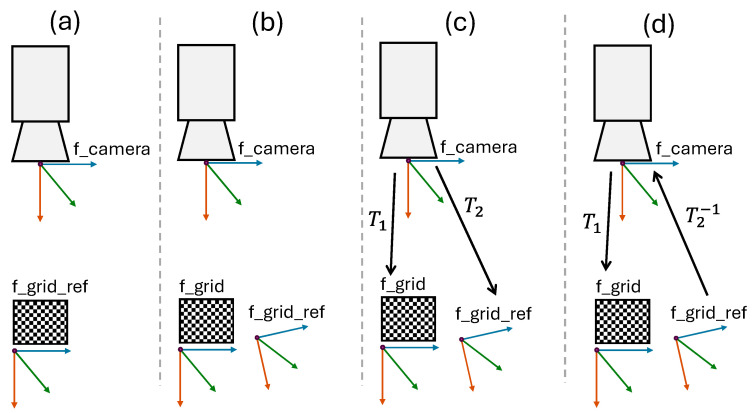
This shows the vision system (**a**,**b**), and the transformation between the camera and the asymmetric circle grid (**c**,**d**).

The camera that is attached to the robot has a frame $f_{camera}\;$defining its location, as shown in Figure 5. The camera frame is virtual, meaning you cannot simply measure its location by a physical measurement. When a process module is initially installed and calibrated in regard to the robot, target locations for moving with the robot are created on the module. At this time, the location of the asymmetric circle grid on the process module is stored by the camera in the frame f_grid_ref. When the process module has been moved, the new location of the circle grid is stored in the f_grid. The difference between the f_grid_ref and f_grid is calculated with the help of the OpenCV library.

Transforming from f_grid to f_camera, and from f_camera to f_grid_ref, the related transformation is expressed in Equation (1) in the mathematical form. All the frame alignments are shown in Figure 5. (a) shows the alignment between the f_camera and the f_grid_ref when the system was initially installed. (b) shows the same module after being moved. Thus, the current location of the circle grid does not correspond with the initial location of the grid, meaning the system is not well calibrated. (c) shows the $T_{1}$, the transformation between f_camera and f_grid and $T_{2}$ is the transformation between f_camera and f_grid_ref. (d) shows the difference between the f_grid and f_grid_ref. This is found by multiplying the inverse transformation matrix $T_{2}^{-1}\;\times T_{1}$.

There are multiple types of errors that can occur that our system solves. Errors in the system contain translation errors and rotation errors. Two main types of errors are (1) robot system errors and (2) robot vision system errors. Both error types will affect all tools, including the camera. Error type 1 is due to tool tolerances on the robot, and the repeatability error of the industrial robot used. Error type 2 is due to the camera frame, which depends on how accurately we can describe the relationship between the default tool frame and the camera frame.

Any related deviation between the f_grid_ref and the f_grid frame can be detected by the system. We know the position of the camera, since it is mounted on the robot. The position of the f_grid is then measured by the camera. Since we have stored the original position of the module related to the robot in f_grid_ref, the difference between these values is calculated, and the result we get is the vision system error.

The transformation matrices can be edited easily if a new camera needs to be added. The transformation matrix contains information including the orientation and position of the process module.

### 4.2. Vision System Evaluation

**Figure 6:**
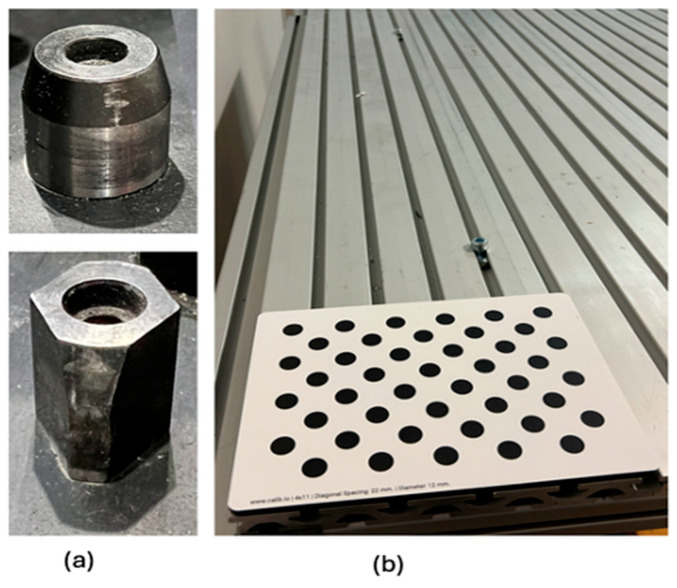
The image on the left (**a**) shows the metal aligners, and the image on the right (**b**) shows the asymmetric circle grid used for calibration, placed on a process module.

To test the accuracy of our system in calibrating process modules, a physical module was used. In Figure 6a, the metal aligner that guides the module to be placed accurately is shown. In Figure 6b, the asymmetric circle grid pattern placed on a process module is shown. The camera uses a RESTful API for communication. The camera sends data variables like $W_{obj}$ and the robot target to the robot controller. The tool data is available in the robot controller.

**Figure 7:**
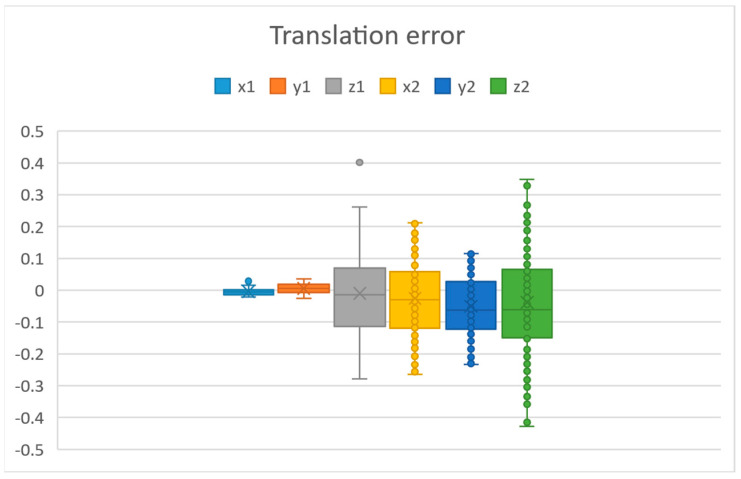
Translation errors in millimetres before and after moving the camera.

**Figure 8:**
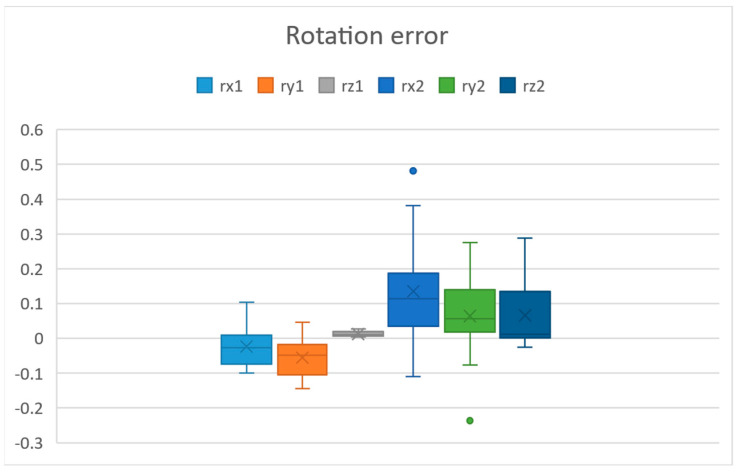
Rotation errors in degrees before and after moving the camera.

In Figure 7, we can see the translation error values in millimetres in all axes, and in Figure 8, we can see the rotation error values in degrees in all axes. The axes x1, y1, z1 and rx1, ry1, rz1 are measured without moving the robot and camera. This corresponds to the error of the vision system. This was measured by fixing the robot and process module, taking multiple measurements. The axes x2, y2, z2 and rx2, ry2, rz2 are the errors after the robot moved the camera.

The vision system error was computed with the help of a calculated reference point, meaning that a calculated reference point in the context of machine vision refers to a specific position or location that is determined based on visual data captured by a camera or imaging system. This reference point is used as a baseline or fixed position for calculating the vision system error. The errors measured after the camera has been moved by the robot are the robot system error. Since we have the correct *f_grid_ref* stored, we do not need to move the process module to find the accuracy of our process module calibration. We can move the camera and observe the error in the vision system. This error will be the accuracy of our complete calibration when inserting a process module. From Figure 7 and Figure 8, we can see that the biggest errors are in z2, $\min\left(z2\right)=\;-0.428097347$ and rx2, max(rx2)= 0.480913962. The standard deviations in each translation axis after the robot moved the camera were x2=0.12251597, y2=0.091317875, z2=0.163471918. These were calculated using Equation (2).

(2) $$\sqrt{\frac{{\sum \left(x-\bar{x}\right)}^{2}}{n-1}\;}$$

For rotation error, we see that the error is around 0.48 degrees. Initially, without camera calibration, we had an error of 1000 microns. If looking individually at the axes, the biggest error in translation was z2, with around 428 microns and an average error of less than 51 microns. This can be further improved using a smaller asymmetric circle grid and moving the camera closer to it. When the robot has located the camera above the circle grid, the time to calibrate is almost instantaneous since it takes one photo. The time to move the camera to the circle grid depends on where the robot TCP is currently located in the system.

## 5. Conclusions and Discussion

This article proposes a novel system for automatically updating a digital twin model with calibration values that is meant for collision detection while performing path planning. The contribution of this article is the proposed system that describes how multiple systems can be connected. It also includes a design and experiment on a camera-based vision system used for calibration. The proposed system defines that by storing data locally on process modules, the system allows them to be moved between stations and carry the information in the module. Physical flexibility and standard connectors make the installation possible in minutes. It is possible to automate the calibration sequence using a camera attached to the robot to determine the process module location each time it is connected to the system. CAD models, calibration values, robot state, and target locations can be automatically updated to a digital twin to avoid manual work on updating simulation models. Path planning should be used to avoid manually creating paths. When a path is generated, the proposed system makes it possible to automatically send this to the robot. This is avoiding manually deploying programs to the robot, which typically requires downtime and long preparation. Using the proposed system, the time to adapt manufacturing to new part requirements and install process modules is significantly decreased. The camera-based vision system was implemented using an asymmetric circle grid attached to the process module and a camera attached to the robot. The results show that if looking individually at the axes, the biggest error in translation was around 428 microns, with an average error of less than 51 microns. In future work, the system can be made more general using a simulation software that supports robot models from multiple manufacturers. Since a process module can carry a smaller robot, it is of interest to investigate in the future how that robot model would be added automatically to the digital twin and included in the path planning. This would allow for faster adaptation to new manufacturing demands.

## Data Availability

The original contributions presented in this study are included in the article. Further inquiries can be directed to the corresponding author(s).
